# The Relationship between Alexithymia, Anxiety, Depression, and Internet Addiction Severity in a Sample of Italian High School Students

**DOI:** 10.1155/2014/504376

**Published:** 2014-10-20

**Authors:** Giuseppe Scimeca, Antonio Bruno, Lucia Cava, Gianluca Pandolfo, Maria Rosaria Anna Muscatello, Rocco Zoccali

**Affiliations:** Department of Neurosciences, University of Messina, Via Consolare Valeria 1, 98125 Messina, Italy

## Abstract

We aimed to assess whether Internet addiction (IA) severity was related to alexithymia scores among high school students, taking into account the role of gender differences and the possible effect of anxiety, depression, and age. Participants in the study were 600 students (ages ranging from 13 to 22; 48.16% girls) recruited from three high schools in two cities from Southern Italy. Participants completed a sociodemographic questionnaire, the Toronto Alexithymia Scale, the Internet Addiction Test, the Hamilton Anxiety Scale, and the Hamilton Depression Scale. The findings of the study showed that IA scores were associated with alexithymia scores, over and above the effect of negative emotions and age. Students with pathological levels of alexithymia reported higher scores on IA severity. In particular, results showed that difficulty in identifying feelings was significantly associated with higher scores on IA severity. No effect of gender was found. Implications for clinicians were discussed.

## 1. Introduction 

There is a growing body of literature showing the emergence of a new category of pathological addictive behavior: Internet addiction (IA) [1–3]. IA refers to the excessive and uncontrollable use of the Internet which leads to several maladaptive consequences such as poor academic and professional performance, relational maladjustment, missed sleep [1, 4–6], and even psychiatric symptoms [7, 8].

Alexithymia is a construct which comprises a pragmatic, externally oriented, and literal cognitive style; a difficulty in recognizing and distinguishing between feelings and bodily sensations and in describing feelings to others; and restricted imagination, marked by the paucity of fantasies, dreams, and daydreaming [8]. Alexithymia has been traditionally reported among patients affected by psychosomatic illnesses, and its role in predicting mind-body associations has been confirmed [9].

Beyond psychosomatic and somatoform disorders, alexithymia has been also associated with addictive disorders, such as pathological gambling [10, 11], substances and alcohol abuse and dependence [12, 13], and eating disorders [14–16]. A possible explanation for this association is that alexithymic individuals may try to self-regulate emotional states through addictive behaviors [17].

Several recent studies have found a relationship between alexithymia and IA among university students [18–21]. Main findings have shown that the Difficulty in Identifying Feelings subscale of the Toronto Alexithymia Scale-20 (TAS-20) was significantly associated with higher risk of IA [19, 20] and that alexithymia partially mediated the association between early experiences of child maltreatment and problematic Internet use [21].

Nevertheless, no studies have addressed the relationship between alexithymia and IA severity in samples of high school students. IA is a growing problem among adolescents: prevalence studies from different European countries reported that up to 4% of adolescents aged from 11 to 18 years have developed a maladaptive use of the Internet [22–24]. In adolescence, IA is associated with difficulties in everyday routine, school performance, and family relationships [25] and with psychiatric symptoms [26]. Thus, expanding knowledge about personality correlates of IA in this susceptible population may allow the development of effective prevention and intervention programs.

There are several reasons to hypothesize an association between alexithymia and IA severity in high school students. Alexithymic adolescents—likewise adults—tended to have difficulties in developing healthy, intimate, and close relationships since alexithymia may interfere with managing emotional states, and it may be related to intense negative emotions, such as anxiety, depression, and anger [27, 28]; separation anxiety; and avoidant tendencies [29]. It has been also suggested that the Internet may be an excellent environment for people who have difficulties in establishing relationships, because of the absence of physical presence and proximity together with the absence of the direct observation of others; these features may allow the Internet communicators to reach greater control over the communication process [30]. Thus, adolescents who have difficulties with identifying, expressing, and communicating emotions may overuse this tool in order to better regulate their emotions and to fulfill their unmet social needs. For this reason, the first hypothesis of this work was that alexithymic tendencies would positively predict Internet addiction severity.

Further, we were interested in examining gender differences. There are reasons to hypothesize that the association between alexithymia and compulsive Internet use may be stronger for men than for women. Women are more skilled with emotional experience [31], and they tend to use more emotion-regulation strategies than men to cope with situations [32]. Also, alexithymia is associated with worse psychological outcomes for men than for women, as suggested by the frequent use of healthcare services found among alexithymic men, but not among alexithymic women [33]. Consistent with these findings, in obese males, alexithymia was more significantly associated with compulsive emotional eating than in obese females [34]. Thus, we examined gender as a potential moderator of these associations, predicting that the effect of alexithymia scores on Internet addiction severity would be affected by gender differences.

A further aim of the present study was to verify whether anxiety and depression symptoms could affect the association between alexithymia and IA severity, since these negative emotions are associated with both alexithymia [35] and IA severity [36]. The effect of age was additionally taken into account, since it has been shown that, in adolescence, both alexithymia and Internet addiction were positively correlated with age [37, 38].

Finally, we were interested in exploring the associations between alexithymia and the different aspects of Internet addiction as measured by the factors identified by the Italian validation of the Internet Addiction Test [39]. Given the lack of previous research, no specific predictions were made: results were examined in an exploratory way by comparing alexithymic subjects with non alexithymic subjects on the different IAT factors.

## 2. Method

### 2.1. Participants

The project was developed for high-school students (from first-year to fifth-year classes), after authorization and consent were obtained from school departments. Data were collected from a sample of 600 students recruited from three high schools in two cities from Southern Italy (Cosenza and Reggio Calabria) through surveys distributed in classrooms. 37 subjects (20 males and 17 females) were excluded because their questionnaires were incomplete. All subjects and their parents gave written consent to participation in this institutional review board-approved study. Minors were asked to take home a letter to their parents explaining the aim of the research, which was described as an investigation into the use of new technologies, and asking parents for permission for their son or daughter to participate; moreover, parents were assured that students' responses would be treated as confidential. Students who agreed to participate and those who received parental permission were involved in a single group-testing session (up to 60 minutes) during which self-report measures and clinician rated scales were administered in each classroom by two trained psychiatrists; teachers remained in the classroom to oversee student discipline but were otherwise uninvolved. Prior to the administration of the survey, the study was reviewed and approved by the local ethics committee and by the participating school districts. Data collection covered six months. The mean age of participants was 16.78  years ± 1.63 (range: 13 to 22); no statistically significant differences between males and females with regard to age were found [*t*(598) = 1.38, *P* = 0.42].

### 2.2. Measures

#### 2.2.1. Sociodemographic Variables and Internet Use

A sociodemographic questionnaire was used to ask participants about their age, sex, school grade, normal residence, siblings, and number of family members. Two questions were specifically related to Internet use: the number of hours spent on the Internet each week and the most frequently used online activity.

#### 2.2.2. Internet Addiction Test

The Italian version of the IAT was used to measure levels of Internet use during the previous month [39, 40]. The IAT consists of 20 items scored on a five-point Likert scale assessing different aspects of Internet use [1]. It takes about 15 minutes and total scores range between 20 and 100. Cut-off scores for the IAT were developed to divide Internet users into minimal (scores: 20 to 39), moderate (40 to 59), and excessive (60 to 100) users according to the severity of their Internet-addictive behavior (total IAT scores) [1]. The Italian translation of the IAT has shown good psychometric parameters of reliability, discriminant, and convergent validity [39–41]. The six-factor solution of the Italian version of the IAT was used; it assesses crucial features of Internet addiction: (1) compromised quality of social life; (2) compromised quality of individual life; (3) compensatory use of the Internet; (4) compromised academic/working career; (5) compromised time control; and (6) excitatory usage of the Internet [39, 40]. The six factors used in this study showed acceptable levels of internal consistency (Cronbach's alpha coefficients between 0.82 and 0.84).

#### 2.2.3. Alexithymia

Alexithymia was measured by using the Italian version of the Toronto Alexithymia Scale (TAS-20) [42]. The TAS-20 consists of 20 items rated on a five-point Likert scale, assessing the different aspects of alexithymia. Individuals are considered as showing pathological levels of alexithymia if their score is 61 or above. Previous studies have shown that the Italian version of the TAS-20 has good internal consistency (Cronbach's alpha of 0.75 and 0.82 in normal and clinical groups, resp.) and test-retest reliability over a three-week interval (*r* = 0.77) [42, 43]. The TAS-20 Scale has a three-factor structure congruent with the concept of alexithymia: difficulty identifying feelings (DIF), difficulty expressing feelings (DEF), and externally oriented thinking (EOT). A confirmatory factor analysis showed the same factor structure as the original English version and adequate internal consistency of the subscales, with *α* coefficients equal to or greater than 0.70 [42]. The Italian validation of the TAS-20 among students showed good psychometric parameters of reliability, discriminant, and convergent validity; confirmatory factor analyses on the questionnaire supported the three-factor structure of the TAS-20 [44]. Internal consistency of the total alexithymia score for our sample was also estimated with Cronbach's alpha; the TAS-20 total score showed a good level of internal consistency, with an alpha value of 0.88.

#### 2.2.4. Negative Emotions

The Hamilton Depression Scale (Ham-D) [45] and the Hamilton Anxiety Scale (Ham-A) [46] were used to assess depression and anxiety symptoms. The Ham-D is a 17-item semistructured interview assessing depressive symptoms. The items are rated on 3- or 5-point scales and scores on the scale can range from 0 to 53, with higher scores indicative of higher levels of depression. Scores ranging from 0 to 7 suggest no or minimal symptoms of depression: scores from 8 to 17 indicate mild depression and from 18 to 25 suggest moderate depression, and scores of 26 and above are associated with severe depression. The validity of the Italian version of the scale is reliable and valid [47]; in the current study, internal consistency of the scale was acceptable (alpha = 0.78).

The Ham-A is a semistructured interview consisting of 14 items, each defined by a series of anxiety symptoms. Each item is rated on a 5-point scale. Scores have a possible range of 0 to 56, with higher scores associated with higher symptoms of anxiety. Scores ranging from 0 to 7 suggest no or minimal symptoms of anxiety, whereas scores of 8 and above indicate the presence of anxiety. Previous research documented high reliability and diagnostic concordance for the Italian version of this scale [48, 49]. Ham-D and Ham-A were chosen because they are two widely used and accepted outcome measures for the evaluation of clinical features of anxiety and depression. The Ham-D and Ham-A were administered by two different raters under a blind condition. The interrater reliability of Ham-D and Ham-A was repeatedly tested during the study period with results ranging from 0.74 to 0.88. Internal consistency of the scale in this study was good (alpha = 0.84). 

## 3. Statistical Analyses

Different Chi-square tests were executed to test possible significant associations between categorical variables. Two MANOVAs were executed to search for possible gender differences among the alexithymia scores and the Internet Addiction Test scores. The association between alexithymia scores and Internet addiction scores was analyzed in two different ways. The total sample was subdivided according to the presence of pathological alexithymia and a MANOVA was executed to search for possible differences in IAT scores. Alexithymia was also treated as a continuous variable and hierarchical regression analyses were conducted to analyze the associations between alexithymia and Internet addiction and the hypothesis concerning gender differences and negative emotions. Statistical analysis was performed with SPSS for Windows 16.0.

## 4. Results

The demographic data and prevalence rate of Internet addiction and alexithymic students are shown in Table 1. According to the IAT cut-off score, 158 students (26.3%) were minimal users, 393 students (65.5%) were moderate users, and 49 students (8.2%) were excessive users. Application of Chi-square tests showed no gender differences in the typology of Internet users (*χ* ^2^ = 0.767,  *d*.*f*. = 2, and *P* = 0.682) and an effect of gender on the kind of Internet activity (*χ* ^2^ = 14.59, *d*.*f*. = 2, and *P* = 0.001), with females preferring online communications (37.7%) and males online games (67.8%). There was no association between Internet activities and the three grades of Internet users (*χ* ^2^ = 7.02, *d*.*f*. = 4, and  *P* = 0.135). Concerning alexithymia, a total of 100 (16.7%) participants were classified into the alexithymic group; 43 (13.8%) alexithymic subjects from the total sample were males and 57 (19.7%) females. No gender differences were found among alexithymic students, (*χ* ^2^ = 3.93, *d*.*f*. = 2, and *P* = 0.140).

Table 2 reports descriptive statistics for alexithymia, Internet addiction, anxiety, and depression symptoms by gender. All scores were in the normal range according to the Italian normative sample [39, 44]. A MANOVA using Pillai's criterion was used to evaluate possible gender differences among the three factors and the total score of the TAS-20; this yielded a multivariate significant effect,* F* (3, 596) = 2.798, *P* < 0.04. Univariate comparisons also yielded significant differences for three scales, with female group reporting higher levels of identifying feelings and externally oriented thinking factors, as well as higher total TAS-20 scores. No gender difference was found for anxiety, depression, and IAT scores. A MANOVA using Pillai's criterion was used to evaluate possible group differences (alexithymic versus nonalexithymic students) among the six factors and the total score of the IAT (Table 3); this yielded a multivariate significant effect,* F* (7, 592) = 3.504, *P* < 0.001. A Bonferroni correction was applied to the MANOVA analysis, and a significance value of *P* < 0.007 was chosen, since seven differences were tested (six factors plus the total IAT score, 0.05/7). Univariate comparisons showed that the alexithymic students reported higher total IAT, compensatory use of the Internet, and compromised time control scores.

Before performing hierarchical regression analyses, multicollinearity was assessed with the variance inflation factor (VIF). VIF scores ranged between 1.32 and 1.66 and the largest condition index (CI) was below 10, suggesting lack of significant multicollinearity [50]. Then, to test our hypotheses, we performed hierarchical multiple regression analysis in which alexithymia variables were entered in Step  1, gender was added in Step  2, and the interaction terms between the alexithymia variables and gender (calculated with partialled products based on centered variables) were added in Step  3.

Table 4 shows the results of the hierarchical multiple regression analysis: alexithymia (expressing feelings and externally oriented thinking) was a significant predictor of Internet addiction scores, accounting for 6% of the variance (Model 1); adding gender in the second step did not significantly increase the explained variance in alexithymia scores (Model 2), as did the interaction between predictors and gender (Model 3).

Finally, to verify whether negative emotions and age affected the association between alexithymia scores and IA severity, a hierarchical multiple regression analysis was performed, adding anxiety, depression, and age on the first step and the alexithymia scale scores on the second step, to examine whether the alexithymia scales would predict IA scores over and above the negative emotions and age variables identified in the first step. Following our predictions (Table 5), the alexithymia scales significantly predicted variance in IA severity scores (Model 2), beyond that predicted by negative emotions and age (Model 1). Adding the alexithymia scales in the second step significantly increased by 3.6% the explained variance in IA scores (Model 2), with difficulty identifying feelings (*sr* = 0.15) significantly contributing unique variance to the prediction of IA severity scores.

## 5. Discussion

The aim of this research was to investigate the association between alexithymia and IA severity in a sample of high school students. The first hypothesis of this study was verified. Our results showed that students with pathological levels of alexithymia reported higher total IAT scores. Furthermore, hierarchical regression analyses showed that alexithymia was a significant predictor of Internet addiction scores, independent from anxiety, depression, and age. Consistent with previous studies [19, 20], our results also suggest that the only TAS-20 factor predicting IA severity was difficulty in identifying feelings. The findings of this study extend previous data showing an association between alexithymia and Internet addiction severity in adult populations [18–21]. There are different possible conceptual explanations for the association between alexithymia and IA severity.

Alexithymic individuals showed difficulty in developing healthy and intimate social relationships because of their inability to correctly identify and manage emotional states [27, 28]. It can be hypothesized that several specific characteristics of the Internet medium may help the alexithymic students to feel more comfortable with relational interactions. Thus, controlling time in terms of preparing messages, choosing when to log on and off, and the chance to rewrite and modify verbal communications may give alexithymic adolescents the opportunity to* better regulate their emotions during social interactions *and to find a more adaptive way to deal with human relationships. It may be useful to evaluate how students experience the ways they relate to others through the Internet to verify whether this variable may act as a moderating or mediating factor in the correlation between alexithymic and Internet addiction scores. Future research may address this question.

Whatever the explanation for this association, our results suggest the importance of assessing possible alexithymic tendencies within high school students with problematic Internet use. Alexithymic trait may be one of the several personality factors that predispose adolescents to the development of IA and hence it should be targeted by psychological approaches aiming to treat IA. Specifically, our results add to the existing literature by showing that alexithymia scores are associated with two different aspects of IA: compensatory usage of the Internet and compromised time control. Taking into account these findings may provide new insight for the behavioral treatment of IA. Psychotherapists who use the cognitive-behavioral model [51] may keep in mind that alexithymic students who develop IA are characterized by an excessive tendency to ruminate about Internet activity; this knowledge might offer new insight for the implementation of cognitive restructuring techniques aimed at reducing rumination. Furthermore, it should be recognized that addressing compromised time control by selecting the duration of use within the behavioral modification plan may influence behavior modification techniques. Future research involving psychotherapeutic treatment of IA may try to verify these hypotheses.

Concerning the role of gender, the hierarchical regression analysis showed that gender did not act as a moderator of the association between alexithymia and Internet addiction. This result is not consistent with findings from adult literature suggesting that alexithymia is more strongly involved in addictive behaviors of men than women [34]. It could be not easy to understand the reason for this unexpected finding, mainly because alexithymia in adolescence remains an understudied topic. It can be hypothesized that, in adolescence, alexithymic females have not yet developed alternative emotion-regulation strategies to cope with emotional situations. The psychometric assessment use of coping styles and emotion-regulation strategies may provide further insight into this hypothesis [32]. Future studies may address this topic.

The current study has several limitations. The research sample consisted of high school students recruited from an urban area of Southern Italy: results would have been more valid if the sample was more heterogeneous with regard to age and cultural/socioeconomic variables. Moreover, the correlational design does not allow drawing firm conclusions, since the possible effect of other third variables cannot be excluded. A further limitation is that agreement has not yet been reached regarding the three-factor structure of the TAS-20 and, specifically, the EOT subscale of the TAS-20 has shown low reliability. Although the TAS-20 has been validated in different adolescent samples [52–54], the three-factor solution of the TAS-20 did not always receive support in adolescents' samples because of age differences in the factor structure [55]; thus, results pertaining to the TAS-20 subscales should be cautiously assumed. Finally, the psychometric assessment was conducted by using self-report instruments: given that alexithymia is per se characterized by a difficulty of self-reflexivity and that self-report measures require self-reflection skills, the validity of our conclusions is not guaranteed. Future research integrating the assessment of alexithymia with observer reports should be needed to replicate these findings.

## 6. Conclusion

IA is a growing problem in the field of pathological addictive behavior. A better knowledge of the relationship between alexithymia and IA may lead to a broader understanding of the etiology and pathogenesis of this addictive behavior, providing new insights into the development of specific psychological and psychosocial approaches aimed at the prevention and treatment of IA. The current study showed that IA scores are associated with alexithymia scores in samples of high school students over and above the effect of depression and anxiety. In particular, results show that difficulty in identifying feelings is significantly associated with higher scores on IA severity. No effect of gender was found.

## Figures and Tables

**Table 1:** Participants sociodemographics by gender.

Factor	Total (*n* = 600)	Males (*n* = 311)	Females (*n* = 289)
	*N* (%)	*N* (%)	*N* (%)
Age (years)			
13-14	55 (9.1)	33 (10.6)	22 (7.6)
15-16	198 (33)	102 (32.8)	96 (33.2)
17-18	250 (41.6)	136 (43.7)	114 (39.4)
19–22	97 (16.1)	40 (12.9)	57 (19.7)
Range	13–22	13–22	14–21
Mean (SD)	16.78 (1.63)	16.69 (1.61)	16.88 (1.66)
Median	17.00	17.00	17.00
School grade			
First	120 (20)	64 (20.6)	56 (19.4)
Second	78 (13)	43 (13.8)	35 (12.1)
Third	135 (22.5)	77 (24.8)	58 (20.1)
Fourth	116 (19.3)	65 (20.9)	51 (17.6)
Fifth	151 (25.2)	62 (19.9)	89 (30.8)
Residence			
Living with parents	588 (98)	304 (97.7)	284 (98.3)
Others	12 (2)	7 (2.3)	5 (1.7)
Only child			
No	524 (87.3)	275 (88.4)	249 (86.2)
Yes	75 (12.5)	36 (11.6)	39 (13.5)
Number of family members			
<3	10 (6.2)	5 (1.6)	5 (1.7)
3	85 (6.3)	40 (12.9)	45 (15.6)
4	318 (53)	174 (55.9)	144 (49.8)
5	141 (23.5)	69 (22.2)	72 (24.9)
6	33 (5.5)	16 (5.1)	17 (5.9)
>6	13 (2.2)	7 (2.2)	6 (2)
Time being spent for Internet use per week	17.68 (10.47)	18.03 (10.82)	17.30 (10.07)
Online activity			
Information search	49 (8.2)	21 (6.8)	28 (9.7)
Online communications	188 (31.3)	79 (25.4)	109 (37.7)
Online games	363 (60.5)	211 (67.8)	152 (52.6)
Internet users			
Minimal	158 (26.3)	79 (25.4)	79 (27.3)
Moderate	393 (65.5)	204 (65.6)	189 (65.4)
Excessive	49 (8.2)	28 (9.0)	21 (7.3)
Alexithymic adolescents	100 (16.7)	43 (13.8)	57 (19.7)

**Table 2:** Descriptive statistics of alexithymia, Internet addiction, anxiety, and depression test scores by gender.

	Total (*n* = 600)	Males (*n* = 311)	Females (*n* = 289)	*P* ^a^
	M (SD)	M (SD)	M (SD)	
TAS-20	54.99 (11.12)	53.81 (11.18)	56.27 (10.93)	0.007
Identifying feeling	16.47 (5.78)	16.00 (5.47)	16.98 (6.07)	0.037
Expressing feelings	13.33 (3.62)	13.11 (3.66)	13.57 (3.58)	0.126
Externally oriented thinking	25.18 (4.91)	24.69 (5.26)	25.71 (4.45)	0.037
IAT	47.82 (12.36)	48.43 (12.46)	47.17 (12.23)	0.215
Ham-A	3.55 (1.51)	3.43 (0.84)	3.68 (0.91)	0.161
Ham-D	3.45 (1.52)	3.36 (0.87)	3.55 (0.89)	0.303

TAS-20: Toronto Alexithymia Scale, IAT: Internet Addiction Test, Ham-A: Hamilton Anxiety Scale, and Ham-D: Hamilton Depression Scale.

^a^Multivariate and univariate analyses with GLM.

**Table 3:** Descriptive statistics of internet addiction test scores by alexithymia.

	Total (*n* = 600)	Nonalexithymic individuals (*n* = 500)	Alexithymic individuals (*n* = 100)	*P* ^a^
	M (SD)	M (SD)	M (SD)	
IAT	47.82 (12.36)	46.93 (11.75)	52.30 (14.26)	<0.000
Compromised social quality of life	16.03 (4.47)	15.75 (4.26)	17.43 (5.17)	0.001
Compromised individual quality of life	30.29 (3.32)	30.14 (3.26)	31.06 (3.54)	0.012
Compensatory usage of the Internet	7.25 (2.68)	7.08 (2.59)	8.11 (2.98)	<0.000
Compromised academic/working careers	2.62 (1.26)	2.57 (1.26)	2.90 (1.25)	0.017
Compromised time control	4.88 (1.83)	4.75 (1.73)	5.56 (2.13)	<0.000
Excitatory usage of the Internet	4.41 (1.97)	4.34 (1.83)	4.76 (2.01)	0.052

TAS-20: Toronto Alexithymia Scale and IAT: Internet Addiction Test.

^a^Multivariate analysis with GLM.

**Table 4:** Hierarchical multiple regression analyses predicting internet addiction severity from gender.

	Model 1					Model 2					Model 3				
	*β*	*t*	*P*	*r*	*sr*	*β*	*t*	*P*	*r*	*sr*	*β*	*t*	*P*	*r*	*sr*
Subscale expressing feelings	−0.23	−3.44	0.001	0.07	−0.13	−0.23	−3.49	0.001	0.07	−0.14	−0.24	−3.61	0.000	0.07	−0.16
Subscale externally oriented thinking	−0.27	−4.13	0.000	0.04	−0.16	−0.26	−4.08	0.000	0.04	−0.16	−0.27	−4.16	0.000	0.04	−0.16
Sum score TAS-20	0.53	5.86	0.000	0.16	0.23	0.54	5.94	0.000	0.16	0.24	0.55	6.06	0.000	0.16	0.24
Gender						0.07	1.68	0.094	0.05	0.07	−0.16	−0.725	0.469	0.05	−0.03
Interaction											0.32	1.58	0.115	0.06	−0.06
Model *R* ^2^	0.060					0.064					0.068				
*R* ^2^ Change	0.060					0.004					0.004				
*F*	(4,595) = 9.458∗					(5,594) = 8.152∗					(6,593) = 7.226∗				

^*^ *P* < 0.001.

Gender with male coded as 1 and female coded as 2.

**Table 5:** Hierarchical multiple regression analyses predicting IA severity scores from alexithymia scores controlling for negative emotions and age.

	Model 1					Model 2				
	*β*	*t*	*P*	*r*	*sr*	*β*	*t*	*P*	*r*	*sr*
Age	0.00	−0.01	0.999	−0.01	0.01	−0.03	−0.64	0.521	−0.01	−0.03
Ham-D	0.13	2.27	0.024	0.16	0.09	−0.02	−0.13	0.898	0.16	−0.01
Ham-A	0.03	0.47	0.639	0.13	0.02	0.03	0.57	0.570	0.13	0.02
Sexual identifying feelings						0.27	3.59	0.000	0.24	0.15
Subscale expressing feelings						−0.06	−0.98	0.326	0.07	−0.04
Subscale externally oriented thinking						−0.04	−0.55	0.583	0.04	−0.02
Model *R* ^2^	0.02					0.06				
*R* ^2^ Change	0.025					0.036				
*F*	(3.596) = 5.021∗∗∗					(6.593) = 6.342∗∗∗∗				

∗∗∗ *P* < 0.005. ∗∗∗∗*P* < 0.001.
